# Phase angle as a novel indicator of sarcopenic obesity in patients undergoing hemodialysis

**DOI:** 10.3389/fnut.2025.1684789

**Published:** 2025-11-06

**Authors:** Linghong Cheng, Liyang Chang, Ruchun Yang, Rongrong Tian, Manting Xiong, Hongmei Zhang

**Affiliations:** 1 Department of Nephrology, Hangzhou TCM Hospital Affiliated to Zhejiang Chinese Medical University, Hangzhou, Zhejiang, China; 2 School of Nursing, Shanghai Jiao Tong University, Shanghai, China

**Keywords:** sarcopenic obesity, nutritional status, phase angle, bioimpedance spectroscopy, hemodialysis

## Abstract

**Background:**

The relationship between phase angle (PhA) and sarcopenic obesity (SO) in patients undergoing hemodialysis (HD) has not been well established. Therefore, this study aimed to evaluate the relationship between PhA and SO in patients undergoing HD and to determine the cutoff value of PhA that can predict SO.

**Methods:**

We conducted a cross-sectional study of 436 patients undergoing HD. The PhA was measured by bioelectrical impedance analysis. SO was diagnosed according to the revised definition of the Asian Working Group for Sarcopenia combined with obesity based on body fat percentage. The association between PhA and SO was assessed using multinomial logistic regression. The PhA cutoff values for SO were determined using receiver operating characteristic (ROC) curve analysis.

**Results:**

Among the participants, 119 (27.3%) had SO. After adjusting for various confounders, PhA was significantly associated with a lower SO risk [odds ratio (OR) = 0.098, 95% confidence interval (CI): 0.048–0.200]. Furthermore, PhA showed a stronger association with SO than with sarcopenia or obesity alone. ROC analysis indicated excellent predictive ability for SO in both sexes (area under the curve (AUC): 0.841 for males, 0.836 for females; cutoff values: 4.49° for males, 4.18° for females).

**Conclusion:**

PhA exhibited good accuracy in detecting SO in patients undergoing HD, suggesting its utility as a reliable screening tool for the early identification of at-risk individuals.

## 1 Introduction

Sarcopenic obesity (SO) is characterized by the coexistence of sarcopenia and excess adiposity. SO is recognized as an emerging public health concern worldwide (1). The adverse clinical consequences of SO are considered extremely important and more severe than those of sarcopenia or obesity alone (2). Highlighting its significance, a joint consensus statement from the European Society for Clinical Nutrition and Metabolism and the European Association for the Study of Obesity underscored the urgent need for standardized diagnostic criteria (1). The pathophysiology of SO involves a detrimental interplay between adipose tissue expansion and muscle wasting. Key mechanisms include chronic inflammation, insulin resistance, hormonal alterations, and ectopic fat infiltration. These pathways collectively drive muscle protein breakdown and impair anabolic signaling (3). These mechanisms are highly relevant in patients with chronic kidney disease, who exhibit accelerated aging phenotypes due to uremic toxins, chronic inflammation, and hormonal imbalances (4, 5). In patients with end-stage renal disease (ESRD), the most common body composition trajectory over time is an increase in body fat accompanied by a loss of lean mass (6–8). Consequently, the impact of SO on patients undergoing dialysis has garnered increasing attention. The prevalence of SO has been reported to vary among studies because of the differences in the definition, diagnostic tools, and study population; however, it is thought to be 16–22% in hemodialysis (HD) patients (9–11), a prevalence notably higher than that reported in the general elderly population (8–11%) (12, 13). In the HD population, SO is independently associated with poor prognosis, including poor gait performance, weakness, decreased quality of life, increased risk of cardiovascular events, and high mortality (10, 14–17). However, early stages of SO are frequently undetected, leading to delays in diagnosis and treatment. Therefore, implementing routine screening for SO in patients undergoing HD is crucial to facilitate early detection and intervention, thereby preventing disease progression.

The phase angle (PhA), a parameter derived from bioelectrical impedance analysis (BIA), is calculated as the ratio of reactance (Xc) to resistance (R). Xc is the membrane’s ability to sustain electrical potential, and R is the opposition offered by body fluids and electrolytes. Consequently, PhA provides information on tissue hydration, cell membrane mass, cellular health, membrane integrity, and cellular function (18). Notably, PhA may serve as a low-cost, real-time alternative for assessing inflammatory status. During states of inflammation and oxidative stress, reactive oxygen species disrupt cell membranes and disturb the intracellular-extracellular fluid balance, which in turn impairs membrane capacitance and thereby lowers PhA (19, 20). Consequently, lower PhA values are increasingly recognized as markers of systemic inflammation and oxidative stress—core mechanisms linking sarcopenia and obesity (21). Given these properties, PhA has been proposed as an independent predictor of disease severity in conditions such as malnutrition (22), nutritional risk (23), and sarcopenia (24). However, its utility for identifying SO in the HD population remains systematically unexplored.

Given that SO is common among patients undergoing HD and seriously endangers their health, a better understanding of the relationship between SO and PhA may spark interest in the nephrological community. Therefore, we conducted this cross-sectional study to investigate the association between PhA and SO in a Chinese HD cohort and to establish the optimal cutoff values of PhA for identifying SO.

## 2 Methods

### 2.1 Study design and participants

We conducted this cross-sectional study between December 2018 and September 2020 at the HD center of the Hangzhou Traditional Chinese Medicine Hospital in Hangzhou, China. The study recruited adult patients aged 18–80 years who were on maintenance HD three times per week for at least 3 months. The exclusion criteria were patients who had metallic joint prostheses, implanted defibrillators, or cardiac pacemakers; those diagnosed with malignant tumors, advanced liver dysfunction, acute systemic infections, or severe nutritional deficiencies; and those who had experienced any cardiovascular event, including myocardial infarction or stroke, within 3 months before enrollment. A total of 436 patients undergoing HD were included in the final analysis. To ensure an adequate sample size, we conducted a statistical power analysis. Using the pwr package in R with the current sample size (N = 436), *α* = 0.05, and the observed large between-group effect size (Cohen’s *f* = 1.299), the statistical power of this study reached 99%. Furthermore, a reverse power calculation indicated that with 80% power and *α* = 0.05, this study would be sufficient to detect an effect size as small as 0.292 (Cohen’s *f*). Together, these results demonstrate that the sample size of this study is adequate to reliably detect significant effects present in the model (25). This study was conducted in accordance with the ethical principles of the Declaration of Helsinki and approved by the Institutional Ethics Committee of Hangzhou Traditional Chinese Medicine Hospital (No. 2018SQ119). Written informed consent was obtained from all participants prior to enrollment in the study.

### 2.2 Patient characteristics

Demographic characteristics, etiology of renal failure, comorbidities, and dialysis data were systematically extracted from the patients’ medical records at the time of study enrollment. Residual renal function was defined as a 24-h urine output exceeding 200 mL. Laboratory tests were performed on fasting blood samples collected within 1 month of enrollment. Routine hematological and biochemical parameters, including high-sensitivity C-reactive protein (hs-CRP), hemoglobin, albumin, total serum cholesterol, triglycerides, blood urea nitrogen, serum creatinine, serum phosphate, and serum calcium, were measured. Dialysis efficiency was assessed by calculating Kt/V using a single-pool urea kinetic model.

### 2.3 Diagnosis of SO

SO, which is distinguished by the simultaneous presence of sarcopenia and obesity, was thus defined. Sarcopenia was assessed using handgrip strength (HGS) and skeletal muscle mass index (SMI) in accordance with the Asian Working Group’s revised definition of sarcopenia (AWGS 2019). According to these criteria, sarcopenia was diagnosed in patients with low muscle mass (SMI < 7 kg/m^2^ for men and <5.7 kg/m^2^ for women) and low muscle strength (HGS < 28 kg for men and <18 kg for women) (26). Obesity was identified based on the criteria of possessing a body fat percentage (BFP) ≥ 25% in men and ≥35% in women, values which are routinely applied to patients with chronic kidney disease (9, 11, 27). BFP was derived by dividing fat mass (kg) by body weight (kg), while SMI was derived by calculating appendicular skeletal muscle mass (ASM) in kilograms as a function of height squared (m^2^). Participants were subsequently classified into the following categories: sarcopenia without obesity (sarcopenia group), no sarcopenia with obesity (obesity group), SO group, and normal group.

### 2.4 BIA measurements

Participants’ body composition was evaluated using a whole-body bioimpedance spectroscopy device (body composition monitor [BCM], Fresenius Medical Care, Bad Homburg, Germany). The BCM system operates by applying alternating currents at 50 different frequencies (ranging from 5 to 1,000 kHz) and measuring the impedance for each. This device employs a three-compartment model, which presupposes a constant hydration factor for both lean tissue mass and fat tissue mass, and excessive extracellular water was classified into a distinct compartment referred to as “overhydration.” This methodological approach offers theoretical advantages for muscle and fat mass assessment, as it minimizes the confounding effects of hydration status on body composition measurements. Measurements were performed before the HD session by a qualified and experienced dialysis nurse who placed four standard electrodes on the patient while supine: two on each hand and foot, positioned on the side opposite to the vascular access. To ensure consistency and minimize variability, all tests were performed by the same operator in strict accordance with the manufacturer’s instructions. PhA, lean tissue mass, fat mass, overhydration, and total body water were obtained directly through BCM. ASM was determined using Ting-Yun Lin’s prediction equation: ASM (kg) = −1.838 + 0.395 × total body water (L) + 0.105 × body weight (kg) + 1.231 × male sex −0.026 × age (years) (*R*^2^ = 0.914, standard error of estimate = 1.35 kg). This equation was specifically developed and validated in an Asian HD cohort and demonstrated excellent agreement with dual-energy X-ray absorptiometry-derived ASM, exhibiting a minimal mean bias of only 0.098 kg in the validation group (28).

### 2.5 Muscle strength measurement

Muscle strength was assessed using an electronic HGS meter (Guangdong Xiangshan Weighing Apparatus Group, Guangdong, China). Patients grasped the meter with the hand on the non-fistula side, with the elbow fully extended. Each test was performed twice, and the highest HGS value was recorded as the result.

### 2.6 Statistical analysis

Descriptive data for continuous variables are expressed as means ± standard deviations or medians (interquartile ranges) based on their distribution, while categorical variables are presented as frequencies (percentages). Patients were classified into four groups: sarcopenia, obesity, SO, and normal.

Group differences were assessed using chi-square tests for categorical variables and one-way analysis of variance (ANOVA) or Kruskal–Wallis tests for continuous variables based on their distribution. For PhA comparisons across the four groups, one-way ANOVA with Bonferroni post-hoc testing was employed. To address multiple testing in these group comparisons, false discovery rate (FDR) correction using the Benjamini–Hochberg procedure was applied, maintaining a 5% false discovery rate.

Multinomial logistic regression models were applied to evaluate the association between PhA, sarcopenia, and obesity status, with PhA as the independent variable. Four sequential adjustment models were used: Model 1: unadjusted; Model 2: adjusted for age, dialysis vintage, and Body Mass Index (BMI); Model 3: additionally adjusted for albumin, serum calcium, phosphate, hs-CRP, triglyceride, serum creatinine, and blood urea nitrogen; Model 4: further adjusted for diabetes and cardiovascular disease. For regression analyses, we applied both FDR and Bonferroni corrections (*α* = 0.017) to account for multiple comparisons across the outcome groups. Variance inflation factors (VIF) were calculated to assess multicollinearity.

Receiver operating characteristic (ROC) curve analysis, with the area under the curve (AUC), was used to identify PhA cutoff values indicating the presence of SO. Sex-specific cutoff points were established using the Youden index, which is defined as the maximum [sensitivity + specificity − 1]. Internal validation through bootstrapping with 1,000 resamples provided 95% confidence intervals for these cutoff values.

All analyses were performed using R software (version 4.3.0). A two-tailed *p*-value of <0.05 was considered statistically significant.

## 3 Results

### 3.1 Participant characteristics

**Figure 1:**
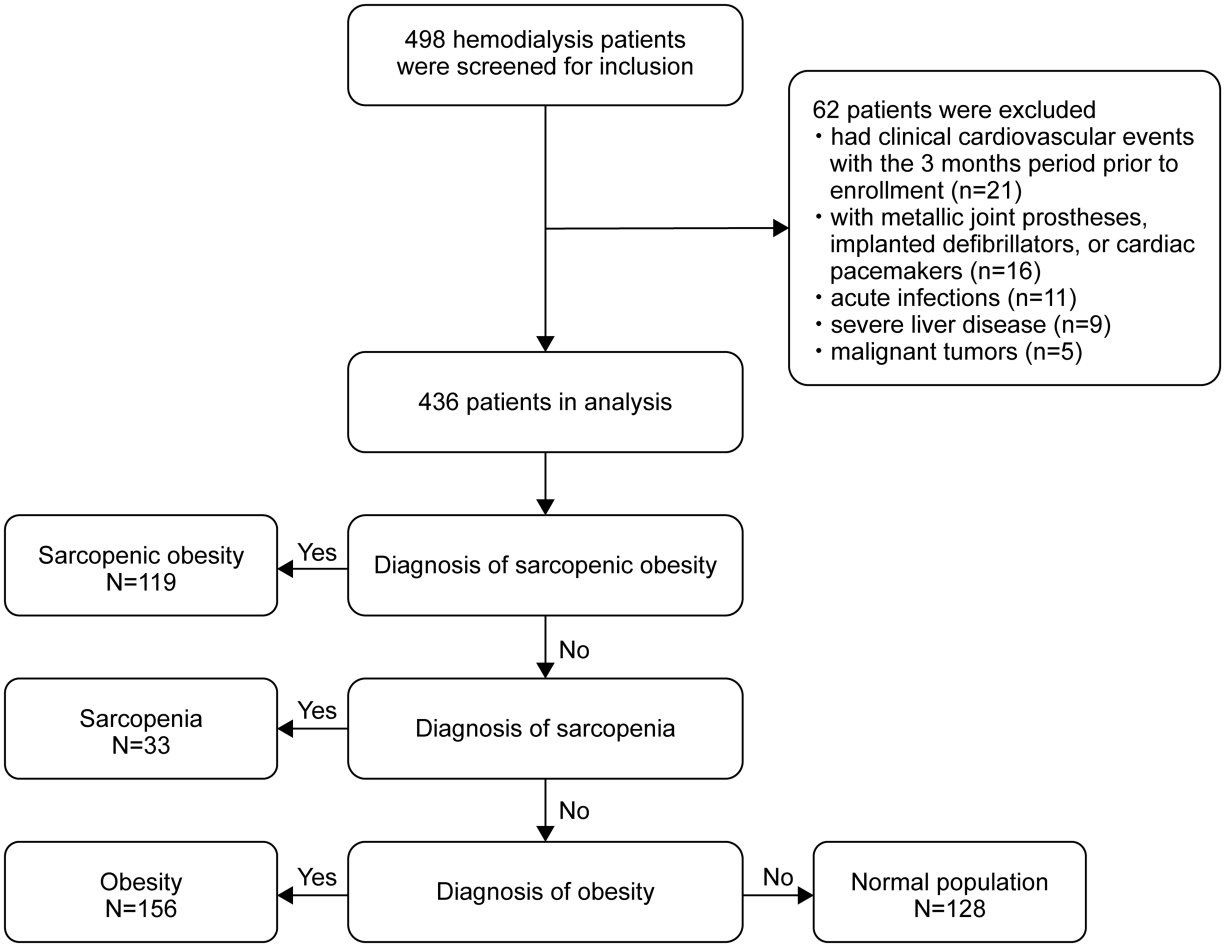
Flow diagram of the study participants.

The flow diagram of this study is shown in Figure 1. Of the 498 patients initially assessed, 436 met the inclusion criteria and were included in the final analysis. The baseline characteristics of the participants according to body composition categories (obese, SO, sarcopenic, and normal) are shown in Table 1. The mean age of the participants was 58.5 ± 13.1 years, and 282 (64.7%) were male. The rates of sarcopenia, obesity, and SO were 7.6%, 35.8%, and 27.3%, respectively. The mean PhA was 4.47 ± 0.90 for all participants, with values of 4.60 ± 0.97 in men and 4.23 ± 0.70 in women. The differences in body weight, body fat, lean tissue mass, BFP, BMI, SMI, and HGS between the groups were in the expected direction. Furthermore, significant differences were found among the four groups in terms of age, sex, dialysis vintage, diabetes and cardiovascular disease, PhA, triglycerides, albumin, hs-CRP, serum phosphate, serum creatinine, and blood urea nitrogen.

**Table 1:** Characteristics of participants by sarcopenia and obesity status.

Variable	Normal population (*n* = 128)	Obesity (*n* = 156)	Sarcopenia (*n* = 33)	Sarcopenic obesity (*n* = 119)	FDR *p*-value
Demographic characteristics					
Age (years)	50.46 ± 11.75	56.22 ± 12.21	66.61 ± 9.39	67.97 ± 8.78	<0.001
Sex, male, *n* (%)	69 (53.9%)	107 (68.6%)	17 (51.5%)	89 (74.8%)	0.002
Residual kidney function, *n* (%)	37 (28.9%)	53 (34.0%)	9 (27.3%)	26 (21.8%)	0.204
Dialysis vintage (m)	61.00 (25.00, 119.50)	46.00 (16.00, 84.00)	80.00 (31.00, 149.50)	49.00 (17.00, 84.00)	0.008
Current smoker, *n* (%)	34 (26.6%)	47 (30.1%)	6 (18.2%)	26 (21.8%)	0.365
Diabetic, *n* (%)	30 (23.4%)	64 (41.0%)	17 (51.5%)	74 (62.2%)	<0.001
Hypertension, *n* (%)	108 (84.4%)	131 (84.0%)	27 (81.8%)	96 (80.7%)	0.630
CVD, *n* (%)	41 (32.0%)	69 (44.2%)	19 (57.6%)	76 (63.9%)	<0.001
Anthropometric measurements and body composition					
Body weight (kg)	54.66 ± 9.63	67.74 ± 11.76	50.24 ± 5.70	58.55 ± 8.70	<0.001
BMI (kg/m^2^)	21.08 ± 2.52	25.45 ± 3.98	19.87 ± 1.68	22.54 ± 2.21	<0.001
OH (L)	2.06 ± 1.27	2.12 ± 1.42	2.54 ± 1.67	2.37 ± 1.31	0.114
Fat mass (kg)	11.93 ± 4.41	24.00 ± 6.62	12.05 ± 3.78	21.72 ± 5.33	<0.001
Body fat percentage (%)	21.82 ± 7.40	35.37 ± 6.97	23.88 ± 6.81	36.97 ± 6.51	<0.001
Lean tissue mass (kg)	38.68 ± 8.48	35.62 ± 8.45	32.98 ± 5.43	28.43 ± 5.89	<0.001
SMI (kg/m^2^)	6.06 ± 1.08	6.67 ± 1.22	5.40 ± 0.68	5.63 ± 0.76	<0.001
HGS (kg)	31.40 ± 9.10	30.96 ± 8.98	18.91 ± 6.19	19.90 ± 6.00	<0.001
PhA (°)	4.93 ± 0.78	4.73 ± 0.84	3.99 ± 0.74	3.76 ± 0.60	<0.001
Laboratory data					
Hemoglobin (g/L)	108.03 ± 12.18	108.98 ± 11.37	108.09 ± 13.01	108.87 ± 13.10	0.842
Total cholesterol (mmol/L)	4.02 ± 0.94	4.03 ± 0.94	4.16 ± 1.11	4.15 ± 1.01	0.767
Triglycerides (mmol/L)	1.60 ± 1.31	2.38 ± 1.54	1.27 ± 0.53	1.91 ± 1.15	<0.001
Serum albumin (g/L)	40.65 ± 2.57	40.28 ± 2.57	38.92 ± 3.77	38.67 ± 4.20	<0.001
Hs-CRP(mg/L)	1.25 (0.58, 2.54)	2.31 (1.25, 5.36)	1.74 (0.60, 4.70)	2.48 (1.11, 6.40)	<0.001
Serum calcium (mmol/L)	2.30 ± 0.19	2.29 ± 0.20	2.31 ± 0.18	2.25 ± 0.21	0.122
Serum phosphate (mmol/L)	1.90 ± 0.49	1.94 ± 0.44	1.76 ± 0.40	1.74 ± 0.47	0.001
Serum creatinine (μmol/L)	935.20 ± 203.89	944.26 ± 219.49	745.36 ± 178.44	755.09 ± 204.20	<0.001
Blood urea nitrogen (mmol/L)	22.04 ± 5.78	23.03 ± 6.15	20.30 ± 5.59	20.90 ± 5.72	0.021

Continuous variables are presented as mean ± SD or median (interquartile range); categorical variables are presented as number (percentage). FDR-adjusted *p*-values were calculated using the Benjamini–Hochberg procedure. CVD, cardiovascular disease; OH, overhydration; Hs-CRP, high-sensitivity C-reactive protein; SMI, skeletal muscle mass index; HGS, handgrip strength; PhA, phase angle.

**Figure 2:**
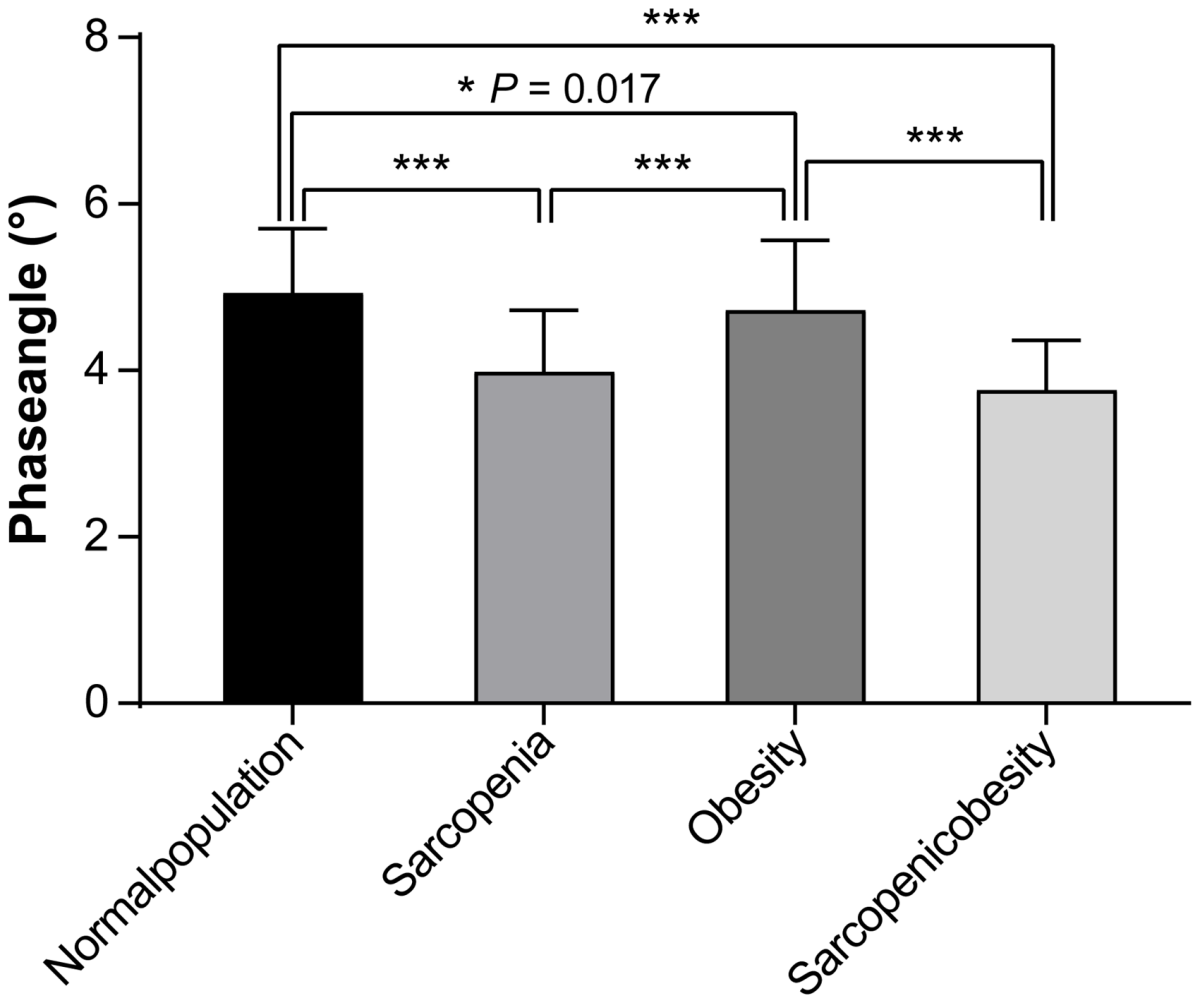
Comparison of the phase angles among the sarcopenia, obesity, sarcopenic obesity, and normal groups.

A comparison of PhA values among the four groups (sarcopenia, obesity, SO, and normal) using one-way ANOVA with a post-hoc test (Bonferroni method) is shown in Figure 2. The PhA values in the sarcopenia, obesity, and SO groups were significantly lower than those in the normal group (all *p* < 0.05). PhA was significantly lower in the sarcopenia group than in the obesity group (3.99 ± 0.74 vs. 4.73 ± 0.8, *p* < 0.001). PhA was significantly lower in the SO group than in the obesity group (3.76 ± 0.60 vs. 4.73 ± 0.8, *p* < 0.001).

### 3.2 The association between PhA and sarcopenia and obesity status

In the multinomial logistic regression, the normal group was employed as the reference, and the relationship between the PhA and SO categories was evaluated (Table 2). In Models 1–3, we observed that the higher the PhA, the lower the risk of sarcopenia, obesity, and SO in all patients. Notably, even in the final adjusted model (model 4), after adjustments for age, dialysis vintage, BMI, albumin, serum calcium, serum phosphate, hs-CRP, triglycerides, serum creatinine, blood urea nitrogen, diabetes, and cardiovascular disease, a higher PhA was still associated with lower risks of obesity (OR = 0.231, 95% CI: 0.127–0.421, *p* < 0.001, *p*_Bonferroni_ < 0.001) and SO (OR = 0.098, 95% CI: 0.048–0.200, p < 0.001, *p*_Bonferroni_ < 0.001), but not with sarcopenia (OR = 0.517, 95% CI: 0.218–1.223, *p* = 0.133, *p*_Bonferroni_ = 0.376) (Supplementary Table S1). Furthermore, the association of PhA with SO was stronger than that with obesity (OR = 0.424, 95% CI: 0.233–0.771, *p* = 0.005). To test whether the association between the PhA and SO categories was stable across sexes, subgroup analyses were performed, and the results were comparable after adjusting for the same variables in Model 4. The VIF values for all models were below 2.14, well below the commonly accepted threshold of 5 (Supplementary Table S2). Model fit and explanatory power were assessed using pseudo R-squared statistics, as shown in Supplementary Table S3. Nagelkerke’s *R*^2^ value, which is a normalized metric, increased sequentially from 0.330 in the unadjusted model (Model 1) to 0.715 in the fully adjusted model (Model 4).

**Table 2:** Multinomial logistic regression analysis of sarcopenic obesity risk according to phase angle values.

Model	Normal	Obesity		Sarcopenia		Sarcopenic obesity	
	OR (95% CI)	OR (95% CI)	*p-*value	OR (95% CI)	*p-*value	OR (95% CI)	*p*-value
Overall phA (per 1°)							
Model 1	Reference	0.736 (0.550–0.985)	0.039	0.194 (0.107–0.351)	<0.001	0.112 (0.070–0.170)	<0.001
Model 2	Reference	0.331 (0.205–0.534)	<0.001	0.286 (0.135–0.607)	0.001	0.099 (0.056–0.178)	<0.001
Model 3	Reference	0.276 (0.157–0.483)	<0.001	0.406 (0.180–0.912)	0.029	0.095 (0.048–0.186)	<0.001
Model 4	Reference	0.231 (0.127–0.421)	<0.001	0.517 (0.218–1.223)	0.133	0.098 (0.048–0.200)	<0.001
Women phA (per 1°)							
Model 1	Reference	0.603 (0.327–1.113)	0.106	0.192 (0.068–0.542)	0.002	0.053 (0.018–0.158)	<0.001
Model 2	Reference	0.140 (0.047–0.422)	<0.001	0.333 (0.085–1.307)	0.115	0.036 (0.009–0.143)	<0.001
Model 3	Reference	0.094 (0.25–0.351)	<0.001	0.722 (0.156–3.337)	0.677	0.060 (0.011–0.320)	0.001
Model 4	Reference	0.078 (0.019–0.319)	<0.001	0.674 (0.131–3.469)	0.637	0.058 (0.009–0.305)	0.001
Men phA (per 1°)							
Model 1	Reference	0.576 (0.389–0.853)	0.006	0.140 (0.064–0.309)	<0.001	0.091 (0.051–0.161)	<0.001
Model 2	Reference	0.390 (0.217–0.700)	0.002	0.299 (0.108–0.772)	0.013	0.104 (0.052–0.209)	<0.001
Model 3	Reference	0.306 (0.155–0.604)	0.001	0.319 (0.098–1.038)	0.058	0.088 (0.039–0.197)	<0.001
Model 4	Reference	0.210 (0.096–0.458)	<0.001	0.398 (0.103–1.535)	0.181	0.073 (0.029–0.180)	<0.001

Model 1: unadjusted.

Model 2: adjusted for age, dialysis vintage, and BMI.

Model 3: adjusted for the covariates in Model 2 plus serum albumin, serum calcium, serum phosphate, high-sensitivity C-reactive protein, triglyceride, serum creatinine, and blood urea nitrogen.

Model 4: adjusted for the covariates in Model 3 plus diabetes and cardiovascular disease. OR, odds ratio; CI, confidence interval.

**Figure 3:**
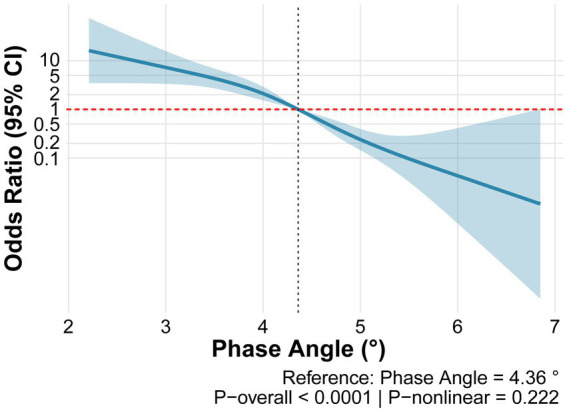
Restricted cubic spline analysis of phase angle and sarcopenic obesity.

Assessment of the dose–response relationship between PhA and SO using restricted cubic spline analysis revealed that the overall model was highly statistically significant (*p* < 0.0001) and demonstrated good discriminative ability (C-statistic = 0.818). Although the nonlinear term did not reach statistical significance (*p*-nonlinear = 0.222), a clear inverse dose–response relationship was observed between PhA and the risk of SO (Figure 3).

To precisely evaluate the independent association between PhA and body composition, Model 5 was constructed by further incorporating Kt/V and overhydration into the fully adjusted model (Model 4). As shown in Supplementary Table S4, after controlling for these two key dialysis-related confounders, the protective associations between PhA and all adverse body composition phenotypes were substantially strengthened: the adjusted odds ratio for SO decreased from 0.098 to 0.008 and for obesity from 0.231 to 0.045, and the association for sarcopenia alone shifted from non-significant to significant, with its odds ratio decreasing from 0.517 to 0.250.

To test for sex differences in the association between PhA angle and body composition phenotypes, we included an interaction term (PhA * sex) in the fully adjusted model. As shown in Supplementary Table S5, the sex interaction effect did not reach statistical significance for any of the body composition phenotypes (all *p* > 0.05). This indicates that the association between the PhA and body composition is consistent across the sexes.

Across all three dialysis vintage subgroups (short: <1 year, medium: 1–3 years, long: >3 years), PhA demonstrated significant inverse associations with SO, with highly consistent effect sizes (OR = 0.152, 0.158, and 0.173, respectively) (Supplementary Table S6).

### 3.3 ROC analysis for PhA to identify patients at risk of SO

**Figure 4:**
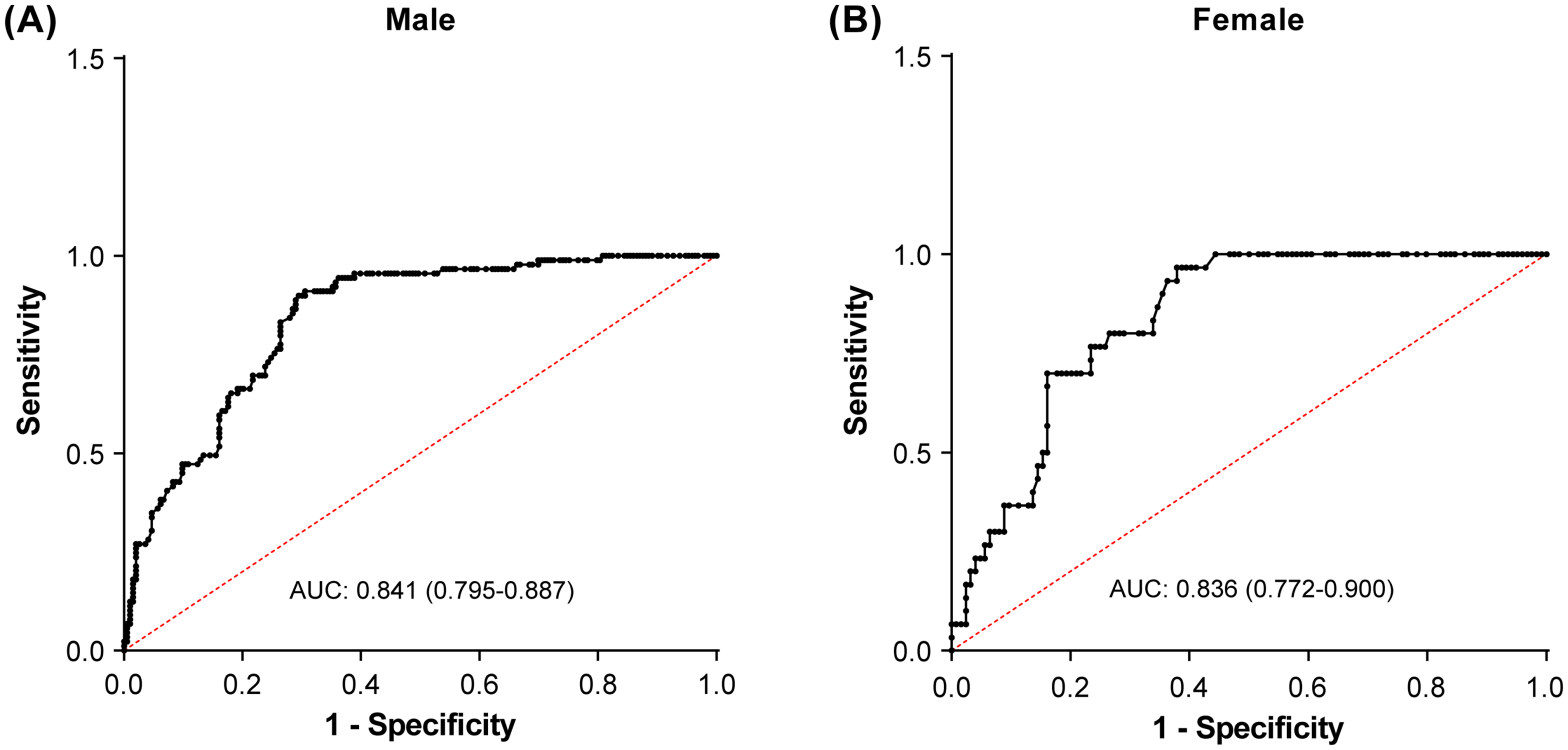
Receiver operating characteristic curves to identify the optimal phase angle cutoff values for detecting sarcopenic obesity in male **(A)** and female **(B)** participants.

Results of the ROC analyses are shown in Figure 4 and Table 3. PhA demonstrated moderate predictive accuracy for SO in all (AUC = 0.818, 95% CI: 0.777–0.859, *p* < 0.001), male (AUC = 0.841, 95% CI: 0.795–0.887, *p* < 0.001), and female (AUC = 0.836, 95% CI: 0.772–0.900, *p* < 0.001) participants. The cutoff value of PhA to discriminate SO from non-SO was 4.48° for all participants with 92.4% sensitivity and 60.3% specificity, 4.49° for male participants with 89.9% sensitivity, and 70.5% specificity, and 4.18° for female participants with 96.7% sensitivity and 62.1% specificity. To validate the stability of these cutoff values, we performed bootstrap resampling with 1,000 repetitions. The bootstrap means were highly consistent with the original estimates. The narrow bootstrap confidence intervals and minimal bias from the original values confirm the robustness of the proposed PhA cutoff values for identifying SO.

**Table 3:** Predictive ability of phase angle and cutoff values for sarcopenic obesity.

Group	AUC (95% CI)	Cutoff (°)	Sensitivity (%)	Specificity (%)	Youden Index	Bootstrap 95% CI
Overall	0.818 (0.777–0.859)	4.48	92.4%	60.3%	0.527	3.99–4.54
Male	0.841 (0.795–0.887)	4.49	89.9%	70.5%	0.604	4.37–4.81
Female	0.836 (0.772–0.900)	4.18	96.7%	62.1%	0.588	3.71–4.28

AUC, area under the receiver operating characteristic curve; CI, confidence interval.

## 4 Discussion

To our knowledge, this is the first study to investigate the association between PhA and SO in patients undergoing HD. This study reports several major findings. First, PhA was significantly associated with SO, even after multivariable adjustments. Second, PhA was more strongly associated with SO than sarcopenia or obesity alone. Third, the optimal cutoff values of PhA for predicting SO were 4.49° for men and 4.18° for women. These findings suggest that PhA is suitable for SO risk stratification, as it is an objective, easily obtainable, non-invasive, and low-cost indicator that minimizes bias for both patients and evaluators.

According to previous studies, the prevalence of SO is increasing because of the aging population worldwide. Its prevalence ranges from 8 to 10% among older adults (12, 13). In our study, SO was present in 27% of patients with ESRD, a prevalence comparable to that in previous studies on patients undergoing HD (11, 29), but obviously higher than that in older adults. However, sarcopenia is frequently overlooked in obese patients. Many patients have excess adiposity concurrent with sarcopenia (9), a condition often missed by BMI assessment alone, leading to clinical misclassification. Notably, patients with SO face a higher mortality risk compared to those with either obesity or sarcopenia alone (10). These findings highlight the critical need to manage SO and develop convenient, non-invasive, and objective tools for its routine assessment in patients undergoing HD.

PhA is a measure of cell stability estimated and interpreted using BIA and serves as a simple and rapid tool in clinical settings (30). As a raw parameter not derived from specific equations, PhA has been shown to reflect cell membrane structure, cell mass, cellular integrity, and cell function, with higher PhA levels indicating better overall cellular health (31). Age, sex, and BMI constitute some of the most important determinants of PhA in healthy populations. Higher PhA values are typically observed in younger individuals and men, attributable to a more favorable body composition characterized by a lower fat percentage and higher muscle mass (32). The present study also found that PhA decreased with age and was lower in women compared to men. Furthermore, after adjusting for multiple variables, logistic regression analysis in our study showed that a low PhA remained significantly associated with SO in patients undergoing HD, regardless of sex. Similarly, Guida et al. (33) demonstrated that overweight and obese patients exhibited a significantly lower PhA, indicating impaired cellular integrity despite higher adiposity. Ding et al. (34) identified PhA as an independent predictor of sarcopenia in patients undergoing HD. This association is consistently observed in populations with diabetes mellitus (35) and post-stroke status (36). Furthermore, PhA was more strongly associated with SO than with sarcopenia or obesity alone, further expanding our understanding of the clinical value of PhA.

Although the exact mechanisms responsible for the association between low PhA and SO remain unclear, studies have shown that PhA is associated with inflammation (37), muscle mass (32), and physical function (38). Specifically, lower PhA values are closely linked to elevated levels of pro-inflammatory cytokines and oxidative stress markers, which collectively promote muscle protein breakdown via ubiquitin-proteasome activation and impair insulin/IGF-1 anabolic signaling pathways (20, 21). Moreover, PhA has been studied as a prognostic marker in several clinical conditions often linked to obesity, including metabolic dysfunction, insulin resistance, and disability, likely due to alterations in cell size and cell membrane permeability (39). These inflammatory and metabolic disturbances create a state of anabolic resistance, further accelerating muscle loss while promoting ectopic fat infiltration into the skeletal muscle (40). It is important to note that these potential mechanistic pathways, while supported by the existing literature, were not directly assessed in this study.

In patients undergoing HD, typical body composition changes over time include body fat gain accompanied by loss of lean mass (41). Muscle loss reduces the basal metabolic rate, physical activity, and energy expenditure in the body, leading to increased fat storage. Higher fat mass produces an excess of proinflammatory cytokines, which stimulate muscle degradation (14). In addition, obesity-related hormonal disturbances can lead to resistance to growth factors, other hormones, amino acids, and the effects of physical exercise, also known as anabolic resistance, which contributes to sarcopenia (42). Thus, the significant association between low PhA and SO suggests that PhA may serve as an indicator of the underlying vicious cycle involving chronic inflammation, oxidative stress, and anabolic resistance. Therefore, assessing PhA might offer a practical approach for early screening and initial stage management of SO.

PhA is a widely used nutritional assessment tool in conditions such as colorectal cancer, liver cirrhosis, and head and neck cancer (43, 44), and it also reflects nutritional status in ESRD (45). A previous study suggested a PhA cutoff value of 4.6° for detecting protein-energy wasting in patients undergoing dialysis (46). Ding et al. (34) subsequently confirmed that a PhA of <4.67° is an independent risk predictor for patients undergoing dialysis with sarcopenia. Despite studies on the association between PhA and nutritional status, none have explored the threshold of PhA for SO identification in patients undergoing HD. In our study, the optimal PhA cutoff value for SO was 4.48°, which is lower than these values. This lower cutoff value likely reflects more severe conditions and poorer cellular function in patients undergoing HD with SO compared to those with protein-energy wasting or sarcopenia (14, 29). Notably, our cutoff value was higher than that reported for post-stroke patients (4.29° for men and 3.84° for women) (36), underscoring the substantial variation in PhA thresholds across disease contexts. Moreover, the ROC analysis in our study demonstrated that PhA exhibited good discriminative ability for predicting SO (AUC = 0.818) in the overall cohort. The optimal PhA cutoff value was 4.48°, yielding a sensitivity of 92.4% and a specificity of 60.3%. High sensitivity indicates effectiveness in identifying true SO cases and minimizing false negatives, establishing PhA as a highly sensitive screening tool. We therefore propose integrating PhA assessment into routine HD care to enable early SO identification in this high-risk population.

This study had several strengths. First, this study investigated the association between PhA and SO and established optimal PhA cutoff values for SO identification in patients undergoing HD, thus providing valuable tools for improving nutritional assessment and risk stratification. Second, this study indicated a stronger association between PhA and SO than between PhA and sarcopenia or obesity alone in patients undergoing HD. Subgroup analyses and detailed ascertainment of potential confounders increased the reliability of the results. Despite all the research efforts, this study had some limitations. First, as this is a cross-sectional study, the design inherently precludes the inference of causality. Thus, it could only examine the associations between the PhA and SO categories. To establish a temporal sequence and verify our findings, future longitudinal cohort studies or interventional trials are necessary. Second, the focus on a Chinese cohort may limit generalizability, as ethnicity, geography, and population characteristics significantly influence PhA values. Asian populations typically exhibit lower PhA baselines due to differences in body composition and cellular integrity compared to Western groups (47). While our findings provide critical insights into similar East Asian demographics, external validation in multiethnic cohorts is essential to establish universally applicable cutoff values and clinical standards. Third, residual confounding may exist from unmeasured lifestyle factors, such as detailed dietary intake and physical activity levels, which could influence both PhA and body composition outcomes. Finally, we acknowledge that BIA is not the gold standard for body composition assessment compared to computed tomography or magnetic resonance imaging. Nevertheless, it is a noninvasive, economical, portable, and safe method. Bioimpedance measurements are comparable to those of dual-energy X-ray absorptiometry (48). Recently, BIA has been recognized as an ideal tool for assessing body composition in both the general population and in patients undergoing HD (49, 50).

## 5 Conclusion

In summary, this study found that low PhA values were independently associated with an increased risk of SO in both men and women. Additionally, PhA showed moderate accuracy in detecting SO, with cutoff values of 4.49° for men and 4.18° for women. These findings suggest that PhA may be a potential marker for identifying patients undergoing HD at risk for SO, although further studies are needed to confirm its clinical applicability.

## Data Availability

The raw data supporting the conclusions of this article will be made available by the authors, without undue reservation.

## References

[1] Donini LM Busetto L Bischoff SC Cederholm T Ballesteros-Pomar MD Batsis JA . Definition and diagnostic criteria for sarcopenic obesity: ESPEN and EASO consensus statement. Obes Facts. (2022) 15:321–35. doi: 10.1159/000521241, PMID: 35196654 PMC9210010

[2] Seo DH Suh YJ Cho Y Ahn SH Seo S Hong S . Effect of low skeletal muscle mass and sarcopenic obesity on chronic kidney disease in patients with type 2 diabetes. Obesity. (2022) 30:2034–43. doi: 10.1002/oby.23512, PMID: 36062861

[3] Hong S Choi KM. Sarcopenic obesity, insulin resistance, and their implications in cardiovascular and metabolic consequences. Int J Mol Sci. (2020) 21:494. doi: 10.3390/ijms21020494, PMID: 31941015 PMC7013734

[4] Kooman JP Kotanko P Schols AMWJ Shiels PG Stenvinkel P. Chronic kidney disease and premature ageing. Nat Rev Nephrol. (2014) 10:732–42. doi: 10.1038/nrneph.2014.185, PMID: 25287433

[5] Duarte MP Almeida LS Neri SGR Oliveira JS Wilkinson TJ Ribeiro HS . Prevalence of sarcopenia in patients with chronic kidney disease: a global systematic review and meta-analysis. J Cachexia Sarcopenia Muscle. (2024) 15:501–12. doi: 10.1002/jcsm.13425, PMID: 38263952 PMC10995263

[6] Carrero JJ Zawada AM Wolf M Stuard S Canaud B Gauly A . Evolution of body composition and wasting indicators by time of day of haemodialysis. Nephrol Dial Transplant. (2021) 36:346–54. doi: 10.1093/ndt/gfaa253, PMID: 33351922

[7] Ng JK-C Chan GC-K Kam KK-H Tian N Than WH Cheng PM-S . The impact of volume overload on the longitudinal change of adipose and lean tissue mass in incident Chinese peritoneal dialysis patients. Nutrients. (2022) 14:4076. doi: 10.3390/nu14194076, PMID: 36235728 PMC9571726

[8] Parthasarathy R Oei E Fan SL. Clinical value of body composition monitor to evaluate lean and fat tissue mass in peritoneal dialysis. Eur J Clin Nutr. (2019) 73:1520–8. doi: 10.1038/s41430-019-0391-3, PMID: 30647437

[9] Ishimura E Okuno S Nakatani S Mori K Miyawaki J Okazaki H . Significant association of diabetes with mortality of chronic hemodialysis patients, independent of the presence of obesity, sarcopenia, and sarcopenic obesity. J Ren Nutr. (2022) 32:94–101. doi: 10.1053/j.jrn.2021.07.003 34465504

[10] Sabatino A Avesani CM Regolisti G Adinolfi M Benigno G Delsante M . Sarcopenic obesity and its relation with muscle quality and mortality in patients on chronic hemodialysis. Clin Nutr. (2023) 42:1359–68. doi: 10.1016/j.clnu.2023.06.032, PMID: 37418843

[11] Tian M Yuan J Yu F He P Zhang Q Zha Y. Decreased intracellular water is associated with sarcopenic obesity in chronic haemodialysis patients. BMC Geriatr. (2023) 23:630. doi: 10.1186/s12877-023-04357-4, PMID: 37803331 PMC10559522

[12] Rossi AP Urbani S Fantin F Nori N Brandimarte P Martini A . Worsening disability and hospitalization risk in sarcopenic obese and dynapenic abdominal obese: a 5.5 years follow-up study in elderly men and women. Front Endocrinol. (2020) 11:314. doi: 10.3389/fendo.2020.00314, PMID: 32695067 PMC7339917

[13] Gao Q Mei F Shang Y Hu K Chen F Zhao L . Global prevalence of sarcopenic obesity in older adults: a systematic review and meta-analysis. Clin Nutr. (2021) 40:4633–41. doi: 10.1016/j.clnu.2021.06.009, PMID: 34229269

[14] De Oliveira Matos B da Costa Rosa CS Ribeiro HS Marcos NM Losilla MPR Monteiro HL . Obesity phenotypes are, in part, associated with physical activity in diabetic hemodialysis patients. Int Urol Nephrol. (2022) 54:1751–9. doi: 10.1007/s11255-021-03060-w, PMID: 34816362

[15] Tian SL Zhang K Xu PC. Increased prevalence of peripheral arterial disease in patients with obese sarcopenia undergoing hemodialysis. Exp Ther Med. (2018) 15:5148–52. doi: 10.3892/etm.2018.6002 29805542 PMC5952103

[16] Kato A Ishida J Endo Y Takita T Furuhashi M Maruyama Y . Association of abdominal visceral adiposity and thigh sarcopenia with changes of arteriosclerosis in haemodialysis patients. Nephrol Dial Transplant. (2011) 26:1967–76. doi: 10.1093/ndt/gfq652, PMID: 20980356

[17] Martinson M Ikizler TA Morrell G Wei G Almeida N Marcus RL . Associations of body size and body composition with functional ability and quality of life in hemodialysis patients. Clin J Am Soc Nephrol. (2014) 9:1082–90. doi: 10.2215/CJN.09200913, PMID: 24763868 PMC4046730

[18] Martins PC Alves Junior CAS Silva AM Silva DAS. Phase angle and body composition: a scoping review. Clin Nutr ESPEN. (2023) 56:237–50. doi: 10.1016/j.clnesp.2023.05.015, PMID: 37344079

[19] Da Silva BR Gonzalez MC Cereda E Prado CM. Exploring the potential role of phase angle as a marker of oxidative stress: a narrative review. Nutrition. (2022) 93:111493. doi: 10.1016/j.nut.2021.111493, PMID: 34655952

[20] da Silva BR Orsso CE Gonzalez MC JMF S Mialich MS Jordao AA . Phase angle and cellular health: inflammation and oxidative damage. Rev Endocr Metab Disord. (2023) 24:543–62. doi: 10.1007/s11154-022-09775-0 36474107 PMC9735064

[21] Jung UJ. Sarcopenic obesity: involvement of oxidative stress and beneficial role of antioxidant flavonoids. Antioxidants. (2023) 12:1063. doi: 10.3390/antiox12051063, PMID: 37237929 PMC10215274

[22] Player EL Morris P Thomas T Chan WY Vyas R Dutton J . Bioelectrical impedance analysis (BIA)-derived phase angle (PA) is a practical aid to nutritional assessment in hospital in-patients. Clin Nutr. (2019) 38:1700–6. doi: 10.1016/j.clnu.2018.08.003, PMID: 30170780

[23] Ramos da Silva B Mialich MS Cruz LP Rufato S Gozzo T Jordao AA. Performance of functionality measures and phase angle in women exposed to chemotherapy for early breast cancer. Clin Nutr ESPEN. (2021) 42:105–16. doi: 10.1016/j.clnesp.2021.02.007, PMID: 33745563

[24] Ruiz-Margáin A Xie JJ Román-Calleja BM Pauly M White MG Chapa-Ibargüengoitia M . Phase angle from bioelectrical impedance for the assessment of sarcopenia in cirrhosis with or without ascites. Clin Gastroenterol Hepatol. (2021) 19:1941–9.e2. doi: 10.1016/j.cgh.2020.08.066 32890753

[25] Faul F Erdfelder E Lang AG Buchner A. G*power 3: a flexible statistical power analysis program for the social, behavioral, and biomedical sciences. Behav Res Methods. (2007) 39:175–91. doi: 10.3758/bf03193146, PMID: 17695343

[26] Chen LK Woo J Assantachai P Auyeung TW Chou MY Iijima K . Asian working group for sarcopenia: 2019 consensus update on sarcopenia diagnosis and treatment. J Am Med Dir Assoc. (2020) 21:300–307.e2. doi: 10.1016/j.jamda.2019.12.012, PMID: 32033882

[27] Lin TY Liu JS Hung SC. Obesity and risk of end-stage renal disease in patients with chronic kidney disease: a cohort study. Am J Clin Nutr. (2018) 108:1145–53. doi: 10.1093/ajcn/nqy200, PMID: 30321257

[28] Lin TY Wu MY Chen HS Hung SC Lim PS. Development and validation of a multifrequency bioimpedance spectroscopy equation to predict appendicular skeletal muscle mass in hemodialysis patients. Clin Nutr. (2021) 40:3288–95. doi: 10.1016/j.clnu.2020.10.056, PMID: 33190991

[29] Zhou C Zhan L He P Yuan J Zha Y. Associations of sarcopenic obesity vs either sarcopenia or obesity alone with cognitive impairment risk in patients requiring maintenance hemodialysis. Nutr Clin Pract. (2023) 38:1115–23. doi: 10.1002/ncp.11044, PMID: 37525570

[30] Xu Y Xie X Duan Y Wang L Cheng Z Cheng J. A review of impedance measurements of whole cells. Biosens Bioelectron. (2016) 77:824–36. doi: 10.1016/j.bios.2015.10.027, PMID: 26513290

[31] Lukaski HC. Evolution of bioimpedance: a circuitous journey from estimation of physiological function to assessment of body composition and a return to clinical research. Eur J Clin Nutr. (2013) 67:S2–9. doi: 10.1038/ejcn.2012.149, PMID: 23299867

[32] Akamatsu Y Kusakabe T Arai H Yamamoto Y Nakao K Ikeue K . Phase angle from bioelectrical impedance analysis is a useful indicator of muscle quality. J Cachexia Sarcopenia Muscle. (2022) 13:180–9. doi: 10.1002/jcsm.12860, PMID: 34845859 PMC8818694

[33] Guida B De Nicola L Pecoraro P Trio R Di Paola F Iodice C . Abnormalities of bioimpedance measures in overweight and obese hemodialyzed patients. Int J Obes. (2001) 25:265–72. doi: 10.1038/sj.ijo.0801475, PMID: 11410830

[34] Ding Y Chang L Zhang H Wang S. Predictive value of phase angle in sarcopenia in patients on maintenance hemodialysis. Nutrition. (2022) 94:111527. doi: 10.1016/j.nut.2021.111527, PMID: 34896667

[35] Hafızoğlu M Yıldırım HK Öztürk Y Şahiner Z Karaduman D Atbaş C . Assessment of phase angle as a novel indicator for sarcopenic obesity according to the ESPEN/EASO criteria in older adults with diabetes mellitus. Nutrition. (2024) 123:112412. doi: 10.1016/j.nut.2024.112412, PMID: 38554459

[36] Yoshimura Y Wakabayashi H Nagano F Matsumoto A Shimazu S Shiraishi A . Phase angle is associated with sarcopenic obesity in post-stroke patients. Clin Nutr. (2023) 42:2051–7. doi: 10.1016/j.clnu.2023.08.018, PMID: 37677910

[37] Bae E Lee TW Bae W Kim S Choi J Jang HN . Impact of phase angle and sarcopenia estimated by bioimpedance analysis on clinical prognosis in patients undergoing hemodialysis. Medicine (Baltimore). (2022) 101:e29375. doi: 10.1097/MD.0000000000029375, PMID: 35758371 PMC9276136

[38] De Souza Francisco D Moraes IG Brito CP Righetti RF Yamaguti WP. The phase angle cut-off point capable of discriminating hemodialysis patients with reduced exercise tolerance: a cross-sectional study. BMC Sports Sci Med Rehabil. (2024) 16:34. doi: 10.1186/s13102-024-00825-5, PMID: 38308310 PMC10835815

[39] Cancello R Brunani A Brenna E Soranna D Bertoli S Zambon A . Phase angle (PhA) in overweight and obesity: evidence of applicability from diagnosis to weight changes in obesity treatment. Rev Endocr Metab Disord. (2023) 24:451–64. doi: 10.1007/s11154-022-09774-1, PMID: 36484943 PMC9735068

[40] Addison O Marcus RL LaStayo PC Ryan AS. Intermuscular fat: a review of the consequences and causes. Int J Endocrinol. (2014) 2014:309570. doi: 10.1155/2014/309570, PMID: 24527032 PMC3910392

[41] Marcelli D Brand K Ponce P Milkowski A Marelli C Ok E . Longitudinal changes in body composition in patients after initiation of hemodialysis therapy: results from an international cohort. J Ren Nutr. (2016) 26:72–80. doi: 10.1053/j.jrn.2015.10.001 26627050

[42] Tantisattamo E Kalantar-Zadeh K Halleck F Duettmann W Naik M Budde K. Novel approaches to sarcopenic obesity and weight management before and after kidney transplantation. Curr Opin Nephrol Hypertens. (2021) 30:14–26. doi: 10.1097/MNH.0000000000000673, PMID: 33186218

[43] Grundmann O Yoon SL Williams JJ. The value of bioelectrical impedance analysis and phase angle in the evaluation of malnutrition and quality of life in cancer patients—a comprehensive review. Eur J Clin Nutr. (2015) 69:1290–7. doi: 10.1038/ejcn.2015.126, PMID: 26220573

[44] Małecka-Massalska T Mlak R Smolen A Morshed K. Bioelectrical impedance phase angle and subjective global assessment in detecting malnutrition among newly diagnosed head and neck cancer patients. Eur Arch Otorrinolaringol. (2016) 273:1299–305. doi: 10.1007/s00405-015-3626-5, PMID: 25859939

[45] Han BG Lee JY Kim JS Yang JW. Clinical significance of phase angle in non-Dialysis CKD stage 5 and peritoneal Dialysis patients. Nutrients. (2018) 10:1331. doi: 10.3390/nu10091331, PMID: 30235860 PMC6165137

[46] Tan R Liang D Liu Y Zhong X Zhang D Ma J. Bioelectrical impedance analysis–derived phase angle predicts protein–energy wasting in maintenance hemodialysis patients. J Ren Nutr. (2019) 29:295–301. doi: 10.1053/j.jrn.2018.09.001, PMID: 30446269

[47] Gonzalez MC Barbosa-Silva TG Bielemann RM Gallagher D Heymsfield SB. Phase angle and its determinants in healthy subjects: influence of body composition. Am J Clin Nutr. (2016) 103:712–6. doi: 10.3945/ajcn.115.116772, PMID: 26843156 PMC6546229

[48] Thomson R Brinkworth GD Buckley JD Noakes M Clifton PM. Good agreement between bioelectrical impedance and dual-energy X-ray absorptiometry for estimating changes in body composition during weight loss in overweight young women. Clin Nutr. (2007) 26:771–7. doi: 10.1016/j.clnu.2007.08.003, PMID: 17936443

[49] Xu Y Li X Hu T Shen Y Xiao Y Wang Y . Neck circumference as a potential indicator of pre-sarcopenic obesity in a cohort of community-based individuals. Clin Nutr. (2024) 43:11–7. doi: 10.1016/j.clnu.2023.11.006, PMID: 37992633

[50] Yang Y Da J Yuan J Zha Y. One-year change in sarcopenia was associated with cognitive impairment among haemodialysis patients. J Cachexia Sarcopenia Muscle. (2023) 14:2264–74. doi: 10.1002/jcsm.1331 37559425 PMC10570075

