# Supplementary material for: Phase angle as a novel indicator of sarcopenic obesity in patients undergoing hemodialysis
Source: Front Nutr. 2025 Nov 6;12:1684789. doi: 10.3389/fnut.2025.1684789 (PMC12630127; doi:10.3389/fnut.2025.1684789)
Supplement: Supplementary file 1 [file Table_1.docx]

Table S1. Multinomial logistic regression analysis of sarcopenic obesity risk according to phA values.

| Variable | Odds Ratio (95% CI) | P-value | P-value (FDR) | P-value (Bonferroni) |
| --- | --- | --- | --- | --- |
| Model |  |  |  |  |
| Normal | Reference |  |  |  |
| Obesity | 0.736 (0.550–0.985) | 0.039 | 0.039 | 0.117 |
| Sarcopenia | 0.194 (0.107–0.351) | <0.001 | <0.001 | <0.001 |
| Sarcopenic Obesity | 0.112 (0.071–0.179) | <0.001 | <0.001 | <0.001 |
| Model 2 |  |  |  |  |
| Normal | Reference |  |  |  |
| Obesity | 0.331 (0.205–0.534) | <0.001 | <0.001 | <0.001 |
| Sarcopenia | 0.286 (0.135–0.607) | 0.001 | 0.001 | 0.003 |
| Sarcopenic Obesity | 0.099 (0.056–0.178) | <0.001 | <0.001 | <0.001 |
| Model 3 |  |  |  |  |
| Normal | Reference |  |  |  |
| Obesity | 0.276 (0.157–0.483) | <0.001 | <0.001 | <0.001 |
| Sarcopenia | 0.406 (0.182–0.904) | 0.027 | 0.027 | 0.082 |
| Sarcopenic Obesity | 0.095 (0.048–0.186) | <0.001 | <0.001 | <0.001 |
| Model 4 |  |  |  |  |
| Normal | Reference |  |  |  |
| Obesity | 0.231 (0.127–0.421) | <0.001 | <0.001 | <0.001 |
| Sarcopenia | 0.517 (0.218–1.223) | 0.133 | 0.133 | 0.376 |
| Sarcopenic Obesity | 0.098 (0.048–0.200) | <0.001 | <0.001 | <0.001 |

Model 1: unadjusted. Model 2: adjusted for age, dialysis vintage and BMI. Model 3: adjusted for the covariates in Model 2 plus serum albumin, serum calcium, serum phosphate, high-sensitivity C-reactive protein, triglyceride, serum creatinine and blood urea nitrogen. Model 4: adjusted for the covariates in Model 3 plus diabetes and cardiovascular disease. OR, Odds Ratio; CI, Confidence Interval; PhA, Phase angle.

| Table S2. Variance Inflation Factors for Predictor Variables in Multivariable Models. | | | |
| --- | --- | --- | --- |
| **Predictor** | **Model2** | **Model3** | **Model4** |
| Phase angle | 1.54 | 2.04 | 2.04 |
| Age | 1.45 | 1.55 | 1.55 |
| Dialysis vintage | 1.03 | 1.14 | 1.14 |
| BMI | 1.12 | 1.26 | 1.26 |
| Albumin |  | 1.26 | 1.26 |
| Calcium |  | 1.14 | 1.14 |
| Phosphorous |  | 1.43 | 1.43 |
| hs-CRP |  | 1.09 | 1.09 |
| Triglyceride |  | 1.13 | 1.13 |
| Creatinine |  | 2.14 | 2.14 |
| BUN |  | 1.63 | 1.63 |
| Model 2: adjusted for age, dialysis vintage and BMI; Model 3: additionally adjusted for laboratory parameters; Model 4: additionally adjusted for comorbidities. Blank cells indicate variables not included in that model. VIF, variance inflation factor; BMI, Body Mass Index; hs-CRP, High-sensitivity C-reactive protein; BUN, Blood Urea Nitrogen | | | |

Table S3. Model fit statistics (effect size) for the multivariable logistic regression models.

| Model | McFadden R² | Cox-Snell R² | Nagelkerke R² |
| --- | --- | --- | --- |
| Model 1 | 0.142 | 0.305 | 0.330 |
| Model 2 | 0.374 | 0.616 | 0.667 |
| Model 3 | 0.400 | 0.640 | 0.694 |
| Model 4 | 0.421 | 0.659 | 0.715 |

Model 1: unadjusted.

Model 2: adjusted for age, dialysis vintage and BMI.

Model 3: adjusted for the covariates in Model 2 plus serum albumin, serum calcium, serum phosphate, high-sensitivity C-reactive protein, triglyceride, serum creatinine and blood urea nitrogen.

Model 4: adjusted for the covariates in Model 3 plus diabetes and cardiovascular disease.

Table S4. Multinomial logistic regression analysis of SO risk according to PhA values: the role of dialysis adequacy and hydration Status.

| Model | Group (vs Normal) | OR (95% CI) | P-value |
| --- | --- | --- | --- |
| Model 4 | Obesity | 0.231 (0.127–0.421) | <0.001 |
| Model 4 | Sarcopenia | 0.517 (0.218–1.223) | 0.133 |
| Model 4 | SO | 0.098 (0.048–0.200) | <0.001 |
| Model 5 | Obesity | 0.045 (0.018-0.116) | <0.001 |
| Model 5 | Sarcopenia | 0.250 (0.076-0.826) | 0.023 |
| Model 5 | SO | 0.008 (0.003-0.023) | <0.001 |

Model 4: adjusted for the covariates in Model 3 plus diabetes and cardiovascular disease. Model 5 was further adjusted for Kt/V and OH. CI, Confidence Interval; SO, Sarcopenic Obesity; PhA, phase angle; OH, overhydration.

Table S5. Analysis of the Sex Interaction Effect on the Association Between Phase Angle and Body Composition Phenotypes.

| Body Composition Group | Interaction Term | Odds Ratio (95% CI) | P-value |
| --- | --- | --- | --- |
| Obesity | phA:genderMale | 1.775 (0.763–4.132) | 0.183 |
| Sarcopenia | phA:genderMale | 1.193 (0.327–4.360) | 0.789 |
| Sarcopenic Obesity | phA:genderMale | 1.729 (0.562–5.328) | 0.340 |

Note: The model was adjusted for age, dialysis vintage, BMI, serum albumin, serum calcium, serum phosphate, high-sensitivity C-reactive protein, triglyceride, serum creatinine, blood urea nitrogen, diabetes, and cardiovascular disease. The reference group is female.

Table S6. Association between phase angle and sarcopenic obesity stratified by dialysis vintage

| Dialysis Vintage | Sample Size | Case Number | OR Value | 95% CI | P Value |
| --- | --- | --- | --- | --- | --- |
| Long-term (>3 years) | 266 | 69 | 0.173 | 0.102-0.293 | <0.001 |
| Medium-term (1-3 years) | 111 | 33 | 0.158 | 0.074-0.338 | <0.001 |
| Short-term (<1 year) | 59 | 17 | 0.152 | 0.047-0.490 | 0.002 |

Note: The model was adjusted for age, dialysis vintage, BMI, serum albumin, serum calcium, serum phosphate, high-sensitivity C-reactive protein, triglyceride, serum creatinine, blood urea nitrogen, diabetes, and cardiovascular disease. The reference group is female.
